# Sleeping behavior and associated factors during COVID-19 in students at a Hispanic serving institution in the US southwestern border region

**DOI:** 10.1038/s41598-023-38713-6

**Published:** 2023-07-18

**Authors:** Humairat H. Rahman, Zainab Akinjobi, Charlotte Gard, Stuart H. Munson-McGee

**Affiliations:** 1 Department of Public Health Sciences, New Mexico State University, Las Cruces, NM 88003 USA; 2 Department of Economics, Applied Statistics and International Business, New Mexico State University, Las Cruces, NM 88003 USA; 3 Data Forward Analytics, LLC, Principal, Las Cruces, NM 88011 USA

**Keywords:** Public health, Epidemiology, Sleep disorders

## Abstract

Sleep is responsible for maintenance and regulatory functions in human physiology. Insufficient sleep has been associated with cardiovascular disease, weight gain, obesity, inflammation, and morbidity. University students are at high risk under normal circumstances of stress and anxiety due to extracurricular demands, competing pressures on student time, long study hours, and financial concerns. The COVID 19 pandemic has disrupted normal college students’ lives adding stresses such as lost jobs and family responsibilities such as serving as caregivers, which disproportionally affect minority and rural student. This study aimed to assess the correlation of sleep disorders in New Mexico State University students during COVID 19 with selected variates including base demographics (e.g., gender, age, etc.), lifestyle metrics (e.g., employment status, discipline, class, etc.), living arrangements (e.g., housing type, number of children, etc.), alcohol and tobacco use, vaccination status, family COVID status, and family vaccination status. Single- and multi-factor logistic regressions were performed to analyze the data on the students. Qualtrics software was used to collect data on demographics and sleep disorders. R software was used for data analysis. Correlations were found between sleeping less, sleeping more, and disturbed sleep among several covariate categories. For all three responses, being married (sleeping less: OR = 0.342, 95% CI = 0.181–0.642, sleeping more: OR = 0.265, 95% CI = 0.111–0.591; disturbed sleeping: OR = 0.345, 95% CI = 0.182–0.650), frequency of feeling sleepy-very often (OR = 16.87, 95% CI = 6.571–47.434; OR = 8.393, 95% CI = 3.086–25.298; OR = 13.611, 95% CI = 5.409–36.975) and change in diet- quality decreased (OR = 7.304, 95% CI = 3.615–15.270; OR = 5.250, 95% CI = 2.309–12.558; OR = 4.181, 95% CI = 2.145–8.359) were all significant correlated to change in sleeping behavior. Other correlations were found among covariates and sleep changes. Several covariates were determined to be correlated with the effect of COVID-19 on sleeping.

## Introduction

Due to the coronavirus (COVID-19) pandemic, educational institutions were forced to close unexpectedly. This closure led to a change in the way students learned, as traditional in-person methods were no longer feasible. In response, alternative learning systems, including web-based technology and e-learning platforms, were implemented. However, this transition presented several challenges for university students as they navigated the new online learning environment^1^. Many of these changes led to mental health becoming an increasing area of concern in college student health.

University students already face high levels of stress and anxiety due to factors such as extracurricular demands, competing pressures on their time, long study hours, financial concerns, and the isolation resulting from limited physical interaction. However, the COVID-19 pandemic has further intensified these challenges. For instance, many students have lost their jobs due to the economic impact of the pandemic, making it difficult to support themselves financially and adding to their worries about an uncertain future. Additionally, the lack of in-person classes and social activities has heightened feelings of isolation and loneliness among students. Also, some students have had to take on caregiving responsibilities for family members, placing additional burdens on their already demanding schedules. These disruptions, combined with the uncertainties brought about by the pandemic, disproportionately affect minority and rural students, exacerbating the existing inequalities they face^2^. Among students at Texas A&M University, 48.14% showed a moderate-to-severe level of depression and 38.48% showed a moderate-to-severe level of anxiety during the COVID-19 pandemic. Approximately 71.26% of participants noted their stress/anxiety levels increased because of the pandemic. Students have noted several reasons for the increase in mental health concerns including transition and maintenance of online classes, stress from academics, concerns over grades, delayed graduation, the health of friends and family, the uncertainty of the pandemic, fear of contracting COVID-19, finances, and isolation^3^.

Sleep disorders have also been cited as a side effect of the COVID-19 pandemic^4^. Sleep is responsible for the maintenance and regulatory functions of human physiology. Insufficient sleep has been associated with cardiovascular disease, diabetes, weight gain, obesity, inflammation, and mortality^5^. Sleep deprivation has effects on metabolism, hormone regulation, and gene expression. Inadequate sleep due to sleep disorders, chaotic lifestyles characterized by irregular routines, excessive workload, and multiple commitments can disrupt sleep patterns, further contributing to sleep deprivation and threaten health and safety^6^. Sleep duration that is inadequate or disrupted can lead to deficits in waking functions including performance, cognitive speed, accuracy, and working memory. It can cause a toll on physiological well-being, affecting emotional and psychosocial interpretation of events and exacerbating stress levels^6,7^. Among university students, healthy sleep habits are important as habits tend to worsen over time and sleep problems can have significant implications on the onset of mental health problems^8^.

Addressing sleep issues in university students is important due to the large number of undergraduates who experience sleep problems and the mental health and clinically relevant symptoms of psychological distress that have been linked to nighttime sleep disruptions^8^. In one study, 86% of college students reported disruptions in their sleep patterns caused by the COVID-19 pandemic. 17% reported irregular sleep patterns associated with inconsistent timing going to bed and waking up, and 6% reported poor sleep quality^9^. Furthermore, among university students in Milan, Italy, the delay in bedtime and in wake-up were more pronounced in students as compared to staff. Worsening sleep quality and insomnia symptoms were found when comparing before to during COVID-19^4^.

This increase in stress noted in literature from the COVID-19 pandemic has led to other changes in behavior such as increases in substance use^3,10^. Among adults, one study found participants consumed more alcohol and smoked cigarettes since the beginning of the pandemic^10^. New barriers such as loneliness and isolation from friends and partners, feelings of frustration, anxiety, and betrayal, and no longer being able to access counseling services exacerbated psychological symptoms and led to an increase in the risk of suicide and substance abuse^11^.

New Mexico has a diverse population with significant Latino, Native American, and rural populations. The rates of COVID-19 among Native Americans living on reservations were more than 4 times higher than rates in the United States. In New Mexico, 34.5% of all positive COVID-19 tests were among American Indian/ Native American people^12^. Studies have found that Hispanic college students have had particularly elevated levels of stress, anxiety, and depression during the pandemic^2^. New Mexico State University (NMSU) is a public land-grant university in southern New Mexico. This study aimed to analyze the factors correlated due to COVID-19 pandemic on the unique population of NMSU students to understand the changes in sleep patterns in this population of university students.

## Methods

A Qualtrics software questionnaire was administered by the Office of Vice President of Research students and staff at NMSU to collect the data. Inclusion criterion for the study was simply that the student had to be at least 18 years old, enrolled at New Mexico State University and willing to answer the questionnaire, i.e. was self-selected. Faculty and staff were not included in this analysis. Informed consent was obtained from all subjects before they start the questionnaire.

Responses to questions on student demographics, lifestyle changes, depression, and other variables were collected. The questionnaire consists of 49 questions on demographics, twenty COVID-19-related questions, nine depression-related questions, and four sleep disorder questions were asked. The questionnaire was sent to approximately 13,900 students and 3300 staff/faculty. However, our population of interest for this research was students only. We received responses 882 responses in total, of which 478 were students. The sample size was calculated using the sample size calculator on Qualtrics software, which calculated the minimum sample size for the student population to be 374 at a 95% confidence level. It took about 20–30 min for the respondents to complete the survey. This survey was conducted from the 21st of April 2022 to the 17th of May 2022.

An individual who slept less than 6 hours (6 h) per night was categorized as sleeping less. An individual with a sleep duration of 7–8 hours (7-8 h) per night was considered to have a standard/normal sleep duration. An individual who slept more than 9 hours (9 h) per night was categorized as sleeping more. Disturbed sleep was classified as a self-reported change in sleeping hours during COVID-19, a sleep problem diagnosed by doctors, and there was an increase or decrease in daytime sleep duration during the COVID-19 pandemic.

The analysis of changes in sleeping behavior data gathered during the survey was analyzed in a three-step process: (1) data clean-up, (2) single-factor analysis, and (3) multi-factor analysis. The data clean-up consisted of redefining non-responsive (e.g., “Do not wish to respond” and blanks) or missing answers as NA so that the subsequent logit regressions by default would not include the response. The second portion of the clean-up was to recategorize major and age. The factor major was recategorized into disciplines based loosely on the NMSU college structure. The new categories were Agricultural, Consumer, and Environmental Sciences (ACES), Arts, Business, Engineering, Health, Education, and Social Transformation (HEST), Humanities, Sciences, and Others. The latter category was frequently students who listed double majors or workforce development degrees. The factor age was simply converted into different age ranges to increase the number of respondents in each to make the analysis more robust.

The single-factor analysis examined how each of the survey factors was correlated to the changes in sleeping behavior responses. Logit regression analysis was conducted using for each of the changes in sleeping behavior as the response factor and the factors within a single variate as the independent regression factors. The regression parameters, including the confidence intervals, were converted into odds ratios and their corresponding confidence intervals. The p-values were used to determine whether or not the odds ratio was statistically different from 1 at the *α* = 0.05 level.

The multi-factor analysis examined how a variate, when corrected for the demographic variates gender, age, race, and marital status, was correlated with the changes in sleeping behavior responses. This was achieved using a logit regression model in which the independent factors included the demographic variates and another single variate from those in the survey data. The regression parameters, including the confidence intervals, were converted into odds ratios and their corresponding confidence intervals. The p-values were used to determine whether or not the odds ratio was statistically different from 1 at the α = 0.05 level.

For all analyses, the cleaned survey data was analyzed directly without the use of weighting factors or other techniques to try ensuring that the survey respondents matched the NMSU student population. All analyses were conducted in base “R” software using version 1.1.463 and the R functions *chisq.test*, *glm* for the logit regression, *confint* for the confidence intervals, and *summary* to extract the regression coefficients from the results^13^.

### Ethics and inclusion statement

Data for this study, including consent form were collected via online using Qualtrics software. The office of vice president of research at NMSU distributed the survey questionnaire to the students. This study is approved by the New Mexico State University Institutional Review Board (IRB). The IRB approval for this research is 22815. Merranda Romero Marin, Ph.D. (email: mmarin@nmsu.edu), Chair of the IRB committee at NMSU at the time of IRB application, has approved our study's final questionnaire. The survey was performed using NMSU students (male and female) who were 18 years and older, and informed consent was obtained before the start of the survey questionnaire; all methods were performed in accordance with the relevant guidelines and regulations. All the collaborators were local and completed the required training to conduct human subject research as per NMSU policy. The data collection and analysis techniques employed raised no risks pertaining to stigmatization, incrimination, discrimination, animal welfare, the environment, health, safety, security or other personal risks. No biological materials, cultural artifacts or associated traditional knowledge has been transferred out of any country or outside NMSU community. The NMSU clinic information was provided to the participants for medical attention if needed to all the participants in the survey. The survey was conducted online only and was anonymous.

## Results

### Single-factor logit regression analysis

The results of the single-factor, or one-factor-at-a-time, logit regression analysis are summarized in Table 1 where the minimum p-value for the categories of each variate is given. In this table, we interpret the results to mean that if the reported p-value is less than 0.05, then at least one of the categories of the variate is statistically different from the referent category. Conversely, if the value is greater than 0.05 we conclude that the categories of the variate are not statistically different.

**Table 1:** Correlation of the survey variates with changes in sleeping behavior based on single-factor logit regression analysis.

Variate		Sleeping less	Sleeping more	Sleep disturbed
Base demographics	Gender	** < 0.001**	**0.017**	*0.074*
	Age	**0.027**	*0.066*	0.336
	Race/ethnicity	*0.055*	0.122	0.369
	Marital status	**0.001**	**0.002**	**0.001**
Lifestyle	Student employment status	**0.017**	0.423	*0.085*
	Class	0.216	0.160	0.210
	Discipline	0.186	0.103	0.138
	International student	*0.080*	0.513	0.958
	Diagnosed with a sleeping problem	**0.015**	0.363	**0.014**
	Hours slept per day	** < 0.001**	** < 0.001**	**0.008**
	Frequency of feeling sleepy	** < 0.001**	** < 0.001**	** < 0.001**
	Exercise status	0.263	0.797	0.733
	Exercise level	0.618	0.507	0.773
	Balanced diet	**0.002**	0.263	**0.043**
	Change in diet	** < 0.001**	** < 0.001**	** < 0.001**
	BMI category	0.274	0.678	0.218
Living arrangement	Housing type	**0.006**	*0.065*	** < 0.001**
	Number of people living together	*0.051*	0.210	0.188
	Have children or not	**0.015**	0.707	0.105
	Children age 5 or less in household	0.213	0.394	0.586
	Adults age 60 or above in household	0.451	0.454	0.283
	Annual household income	*0.086*	0.207	0.184
Alcohol and tobacco use	Alcohol consumption	0.236	0.862	0.945
	Alcohol consumption frequency	0.315	0.402	**0.029**
	Changes in alcohol consumption	*0.086*	0.141	0.720
	Smoking status	0.235	0.652	0.477
	Tobacco intake frequency	0.343	0.887	0.444
	Smoked at least 100 cigarettes	0.491	0.172	0.550
	Number of days smoked	0.277	0.785	0.388
	Average number cigarettes per day	0.712	0.995	0.692
	Change in smoking behavior	*0.051*	0.116	0.234
Vaccination status	Vaccine hesitant	**0.046**	0.136	0.589
	Vaccination status	0.743	0.408	0.555
	Doses received	*0.055*	*0.068*	**0.046**
Family COVID status	Tested positive for COVID	0.122	0.347	0.439
	Quarantined due to COVID	0.106	0.154	0.413
	Parent(s) had COVID	0.167	0.251	0.476
	Sibling(s) had COVID	0.111	0.250	0.838
	Spouse had COVID	0.139	0.353	0.736
	Child(ren) had COVID	0.371	0.794	0.548
	Grandparent(s) had COVID	*0.064*	0.593	0.599
	Family member died of COVID	*0.095*	0.183	0.305
Family vaccination status	Parent(s) vaccination status	0.140	0.995	0.760
	Sibling(s) vaccination status	0.925	0.757	0.433
	Spouse vaccination status	0.103	0.501	0.137
	Children 5 years old or older vaccination status	0.956	0.525	0.935
	Grandparent(s) vaccination status	0.390	**0.026**	0.220

Variates that are statistically significant at α = 0.05 are bolded while the additional variates that would be considered statistically significant at α = 0.10 are in italics.

Of the 47 variates included in the survey, only 14 of them are correlated with the sleeping behavior in this single-factor analysis. Four of the variates (marital status, hours slept per day, frequency of feeling sleepy, and change in diet) were significantly correlated with all three types of changes in sleeping behavior. An additional four variates (gender, diagnosed with a sleeping problem, a perceived balanced diet, and housing type), were correlated with two of the sleeping behavior changes. Finally, another seven variates (age, student employment status, having had children, alcohol consumption frequency, vaccine hesitancy, number of vaccine doses received, and whether or not the student's grandparents had been vaccinated) were correlated with one of the changes in sleeping behavior.

A more careful examination of the data in Table 1 reveals that several other variates would be significant if a slightly higher level of the critical p-value had been chosen. Had a critical p-value of 0.10 been used, another 8 variates (including race/ethnicity, international student status, number of people in the household, annual household income, changes in alcohol consumption, changes in smoking behavior, a grandparent having had COVID, and whether or not a family member had died from COVID) would have been considered statistically significant. These variates may have not been statistically significant in the current study due to the small sample size of the survey and in future research should be included as possible correlating variates. In the remainder of this section, only the results for those variates with a minimum p-value less than 0.05 are considered further.

### Sleeping less single-factor logit regression

The single-factor logit regression results when the binary response variable is sleeping behavior "unchanged" vs. sleeping "fewer hours per night" are given in Table 2 for the variates which showed a statistically significant correlation at the *α* = 0.05 level in Table 1 These results illustrate which levels of the variates were different from the reference levels. The magnitude and direction of these correlations are given by the odds ratios in the tables in the results sections and within the text below. Odds ratios greater than one indicate a positive correlation while those less than one indicate a negative correlation. The strength of the correlation is indicated by the p-values reported in the text and tables. We have chosen to only discuss correlations with p-values less than 0.05 as being statistically significant.

**Table 2:** Correlation of "sleeping less" and selected variates including the odds ratio (OR), the corresponding lower and upper confidence levels (95% CI), and the p-value from the logit regression.

Variate		Level	Sleeping Less			Logit regression results		
			No	Yes	% yes	OR	(95% CI)	p-value
Base demographics	Gender	Female (reference)	44	128	74.4%	–	–	–
		Male	29	21	42.0%	**0.249**	**(0.127, 0.480)**	** < 0.001**
		Non-binary/non-conforming	1	4	80.0%	1.375	(0.194, 28.042)	0.780
	Age	18–19 (reference)	9	32	78.0%	–	–	–
		20–29	33	80	70.8%	0.682	(0.278, 1.55)	0.379
		30–39	22	27	55.1%	**0.345**	**(0.130, 0.860)**	**0.027**
		40 or older	10	14	58.3%	0.394	(0.127, 1.188)	0.101
	Marital status	Single (reference)	43	117	73.1%	-	-	-
		Married	29	27	48.2%	**0.342**	**(0.181, 0.642)**	**0.001**
		Divorced	2	6	75.0%	1.103	(0.242, 7.762)	0.907
		Other	0	3	100.0%			
Lifestyle	Student employment status	No (reference)	49	100	67.1%	–	–	–
		Yes, I am a graduate assistant	8	11	57.9%	0.674	(0.254, 1.859)	0.431
		Yes, I am a student worker	6	33	84.6%	**2.695**	**(1.117, 7.603)**	**0.041**
		Yes, I am a teaching assistant	8	3	27.3%	**0.184**	**(0.038, 0.674)**	**0.017**
	Diagnosed with a sleeping problem	No (reference)	71	128	64.3%	–	–	–
		Yes	2	23	92.0%	**6.379**	**(1.806, 40.903)**	**0.015**
	Hours slept per day	6 h or less (reference)	20	136	87.2%	–	–	–
		7–8 h	40	15	27.3%	**0.055**	**(0.025, 0.115)**	** < 0.001**
		9 h or more	5	1	16.7%	**0.029**	**(0.001, 0.197)**	**0.002**
	Frequency of feeling sleepy	Not at all (reference)	20	8	28.6%	–	–	–
		Occasionally	37	36	49.3%	2.432	(0.972, 6.569)	0.067
		Very often	16	108	87.1%	**16.870**	**(6.571, 47.434)**	** < 0.001**
	Balanced diet	No (reference)	37	112	75.2%	–	–	–
		Yes	35	41	53.9%	**0.387**	(0.214, 0.695)	**0.002**
	Change in diet	No (reference)	35	23	39.7%	–	–	–
		Yes, quality has decreased	20	96	82.8%	**7.304**	**(3.615, 15.27)**	** < 0.001**
		Yes, quality has increased	18	34	65.4%	**2.874**	**(1.330, 6.386)**	**0.009**
Living arrangement	Housing type	Rent apt or house (reference)	12	42	77.8%	–	–	–
		Share apt or house	8	17	68.0%	0.607	(0.209, 1.809)	0.360
		Own house	24	25	51.0%	**0.298**	**(0.123, 0.691)**	**0.006**
		Other	27	65	70.7%	0.688	(0.304, 1.491)	0.354
	Have children or not	No (reference)	53	112	67.9%	–	–	–
		Yes, 1 child	6	19	76.0%	1.499	(0.589, 4.367)	0.422
		Yes, 2 children	6	13	68.4%	1.025	(0.378, 3.095)	0.962
		Yes, 3 children	7	1	12.5%	**0.068**	**(0.003, 0.400)**	**0.015**
		Yes, more than 3 children	2	6	75.0%	1.42	(0.310, 10.180)	0.678
Vaccination status	Vaccination hesitant	No (reference)	42	64	60.4%	–	–	–
		Yes	29	80	73.4%	**1.81**	**(1.019, 3.252)**	**0.046**

*P*-values that are statistically significant at α = 0.05 are bolded.

The data in Table 2 indicate that for the base demographic variates males are only about ¼ as likely to respond to sleeping less than females (OR = 0.249, CI = 0.127–0.480 where OR is the odds ratio and CI is the confidence interval), students in their 30 s are about 1/3 less likely (OR = 0.345, CI = 0.130–0.860), and married students are also about 1/3 less likely (OR = 0.342, CI = 0.181–0.642).

For the lifestyle variates, those who are student employees are about 2 and a half times (OR = 2.695, CI = 1.117–7.603) more likely to report sleeping less than those who are not employees but, interestingly, those who are teaching assistants are about 1/5 less likely (OR = 0.184, CI = 0.038–0.674) to report sleeping less. This discrepancy could potentially be explained by the more structured schedules and regular work hours that teaching assistant positions typically entail, providing them with better sleep opportunities compared to other employed students.

Students who report having been diagnosed with a sleeping problem are over 6 times (OR = 6.379, CI = 1.806–40.903) as likely to report sleeping less than those who have not.

Students who sleep more than six hours per night are much less likely to report sleeping less (7–8 h, OR = 0.055, CI = 0.025–0.115; 9 h or more OR = 0.029, CI = 0.001–0.197) than those sleeping 6 h or less per night. With respect to the frequency of feeling sleepy, students who report that they "often" feel sleepy are more than 16 times as likely to report getting less sleep (OR = 16.870, CI = 6.571–47.434).

Diet also appears to be correlated with sleeping less; students with a balanced diet are less likely to report sleeping less (OR = 0.387, CI = 0.214–0.695) but those whose diet has changed reported an increase in sleeping less (diet quality decreased OR = 7.304, CI = 3.615–15.270; diet quality increased OR = 2.874, CI = 1.330–6.386).

The living arrangement also seems to be correlated with sleeping less. Students who live in their own homes are less likely to report sleeping less (OR = 0.298, CI = 0.123–0.691) than those who rent as are those who have three children (OR = 0.068, CI = 0.003–0.400).

Finally, students who are vaccine-hesitant are almost twice as likely to report sleeping less than students who are not (OR = 1.810, CI = 1.019–3.252).

### "Sleeping more" single-factor logit regression

The results of the single-factor logit regression for the response "sleeping more" compared to students who reported "no change" in their sleeping behavior are given in Table 3 for the variates that showed a statistically significant effect as reported in Table 1. These results illustrate which levels of the variates were different from the reference levels. Only six of the study's variates are significantly correlated with sleeping more. Of the base demographics, gender, and marital status were correlated with sleeping more with males less likely (OR = 0.393, CI = 0.180–0.829) to report sleeping more than females and married students also less likely (OR = 0.265, CI = 0.111–0.591) to report sleeping more than single students.

**Table 3:** Correlation of "sleeping more" and selected variates including the odds ratio (OR), the corresponding lower and upper confidence levels (95% CI), and the p-value from the logit regression.

Variate		Level	Sleeping more			Logit regression results		
			No	Yes	% yes	OR	(95% CI)	p-value
Base demographics	Gender	Female (reference)	44	54	55.1%	–	–	–
		Male	29	14	32.6%	**0.393**	**(0.18, 0.829)**	**0.017**
		Non-binary/non-conforming	1	1	50.0%	0.815	(0.03, 21.949)	0.887
	Marital status	Single (reference)	43	56	56.6%	–	–	–
		Married	29	10	25.6%	**0.265**	**(0.111, 0.591)**	**0.002**
		Divorced	2	2	50.0%	0.768	(0.087, 6.785)	0.798
		Other	0	1	100.0%	NA	NA	NA
Lifestyle	Hours slept per day	6 h or less (reference)	20	12	37.5%	–	–	–
		7–8 h	40	26	39.4%	1.083	(0.453, 2.657)	0.859
		9 h or more	5	31	86.1%	**10.333**	**(3.323, 37.708)**	** < 0.001**
	Frequency of feeling sleepy	Not at all (reference)	20	7	25.9%	–	–	–
		Occasionally	37	15	28.8%	1.158	(0.41, 3.503)	0.786
		Very often	16	47	74.6%	**8.393**	**(3.086, 25.298)**	** < 0.001**
	Change in diet	No (reference)	35	13	27.1%	–	–	–
		Yes, quality has decreased	20	39	66.1%	**5.25**	**(2.309, 12.558)**	** < 0.001**
		Yes, quality has increased	18	17	48.6%	*2.543*	(1.014, 6.562)	*0.051*
Family vaccination status	Grandparents' vaccination status	No, not all living grandparents vaccinated (reference)	14	5	26.3%	–	–	–
		Yes, all living grandparents vaccinated	38	49	56.3%	**3.611**	**(1.248, 12.134)**	**0.026**

*P-*values that are statistically significant at α = 0.05 are bolded.

Three lifestyle variates (hours slept per day, frequency of feeling sleepy, and change in diet) were correlated with sleeping more. Students who reported sleeping 9 or more hours per day were also more likely (OR = 10.333, CI = 3.323–37.708) to report sleeping more and similarly, those reporting feeling sleepy most frequently were more likely (OR = 8.393, CI = 3.086–25.298) than those who reported not feeling sleepy at all.

Changes in diet, whether the quality of the diet decreased (OR = 5.250, CI = 2.309–12.558) or increased (OR = 2.543, CI = 1.014–6.562), are correlated with increase in sleeping.

The grandparents' vaccination status where having all grandparents vaccinated was correlated with an increase in sleeping more (OR = 3.611, CI = 1.248–12.134).

### Disturbed sleep single-factor logit regression

The results of the single-factor logit regression for the students who reported "disturbed sleep" compared to students who reported "no change" in their sleeping behavior are given in Table 4 for the variates that showed a statistically significant effect as given in Table 1. These results illustrate which levels of the variates were different from the reference levels. Of the base demographics, only marital status was correlated with students reporting disturbed sleep with married students less likely (OR = 0.345, CI = 0.182–0.650) to report having disturbed sleep than single students.

**Table 4:** Correlation of "disturbed sleep" and selected variates including the odds ratio (OR), the corresponding lower and upper confidence levels (95% CI), and the p-value from the logit regression.

Variate		Level	Sleep disturbed			Logit regression results		
			No	Yes	%Yes	OR	(95% CI)	p-value
Base demographics	Marital status	Single (reference)	43	116	73.0%	–	–	–
		Married	29	27	48.2%	**0.345**	**(0.182, 0.65)**	**0.001**
		Divorced	2	7	77.8%	1.297	(0.297, 9.081)	0.753
		Other	0	1	100.0%	NA	NA	NA
Lifestyle	Diagnosed with a sleeping problem	No (reference)	71	126	64.0%	–	–	–
		Yes	2	23	92.0%	**6.480**	**(1.834, 41.561)**	**0.014**
	Hours slept per day	6 h or less (reference)	20	76	79.2%	–	–	–
		7–8 h	40	64	61.5%	**0.421**	**(0.22, 0.787)**	**0.008**
		9 h or more	5	9	64.3%	0.474	(0.145, 1.7)	0.227
	Frequency of feeling sleepy	Not at all (reference)	20	9	31.0%	–	–	–
		Occasionally	37	44	54.3%	**2.643**	**(1.093, 6.805)**	**0.037**
		Very often	16	98	86.0%	**13.611**	**(5.409, 36.975)**	** < 0.001**
	Balanced diet	No (reference)	37	98	72.6%	–	–	–
		Yes	35	51	59.3%	**0.550**	**(0.309, 0.978)**	**0.043**
	Change in diet	No (reference)	35	36	50.7%	–	–	–
		Yes, quality has decreased	20	86	81.1%	**4.181**	**(2.145, 8.359)**	** < 0.001**
		Yes, quality has increased	18	29	61.7%	1.566	(0.74, 3.37)	0.245
Living arrangement	Housing type	Rent apt or house (reference)	12	47	79.7%	–	–	–
		Share apt or house	8	23	74.2%	0.734	(0.263, 2.126)	0.558
		Own house	24	17	41.5%	**0.181**	**(0.072, 0.433)**	** < 0.001**
		Other	27	57	67.9%	0.539	(0.238, 1.167)	0.126
Alcohol and tobacco use	Alcohol consumption frequency	Monthly or less (reference)	18	22	55.0%	–	–	–
		Two to four times a month	12	41	77.4%	**2.795**	**(1.138, 7.104)**	**0.029**
		Two to three times a week	6	14	70.0%	1.909	(0.616, 6.46)	0.277
		Four or more times a week	3	8	72.7%	2.182	(0.529, 11.429)	0.307
		Never	1	0	0.0%	NA	NA	NA
Vaccination status	Doses received	1 dose (reference)	5	2	28.6%	–	–	–
		2 doses	11	26	70.3%	5.909	(1.081, 46.73)	0.054
		3 doses	49	110	69.2%	**5.612**	**(1.153, 40.86)**	**0.046**

*P*-values that are statistically significant at α = 0.05 are bolded.

For the lifestyle variates, current sleeping habits and diet were correlated with disturbed sleep. Students who had been diagnosed with a sleeping problem (OR = 6.480, CI = 1.834–41.561) and those who occasionally (OR = 2.643, CI = 1.093–6.805) or very often (OR = 13.611, CI = 5.409–36.975) felt sleepy reported an increase in disturbed sleep while students who slept 7–8 h per day were less likely (OR = 0.421, CI = 0.220–0.787) to report disturbed sleep.

Students who reported having a balanced diet were also less likely (OR = 0.550, CI = 0.309–0.978) to have disturbed sleep while students reporting having their diet changed for the worse were more likely (OR = 4.181, CI = 2.145–8.359) to report disturbed sleep.

Students living in their own house were less likely (OR = 0.181, CI = 0.072–0.433) than students living in a rented apartment or house to report having disturbed sleep. Students who consumed alcohol two to four times a month were more likely (OR = 2.795, CI = 1.138–7.104) to report disturbed sleep than students who drank monthly or less. Finally, fully vaccinated students (i.e. those who had received 3 vaccine doses) were more likely (OR = 5.612, CI = 1.153–40.860) than students with single vaccine dose to report having disturbed sleep.

### Multi-factor logit regression analysis

Multi-factor logit regression was performed to understand how the base demographic variables gender, age, race, marital status, and one other variate were correlated with the change in sleeping behavior response variables. In other words, the basic regression model included those four variates plus the remaining variates selected on a one-at-a-time basis. This analysis is used to "correct" the correlation of the variate with the demographic variables. This can be important when a variate is correlated with one of the base demographic variates; including both of them in the regression model should result in only the one most strongly correlated with the response being statistically significant.

Given in Table 5 are the *minimum* p-values for each variate in the survey when corrected for gender, age, race, and marital status using multi-factor logit regression. Since the respondents to the survey were self-selected, it was not possible to perform this analysis for all of the variates due to a lack of a sufficient number of respondents in each category of all the factors. For five of the variates, the regression model was singular and could not be evaluated. These five variates were the number of children under the age of 5 in the household, whether or not the respondent smoked 100 or more cigarettes in their lifetime, the number of days they had smoked, and the average number of cigarettes smoked per day. Of the remaining 39 variates, only one (hours slept per day) was correlated with all three sleeping response factors (sleeping less, sleeping more, and disturbed sleeping) at the α = 0.05 significance level. Three of the variates (diagnosed with a sleeping problem, frequency of feeling sleepy, and housing type) were correlated with two of the response factors at the *α* = 0.05 significance level. Four variates (balanced diet, number of people living together, changes in alcohol consumption, and tobacco intake frequency) were correlated with one of the responses at the *α* = 0.05 significance level.

**Table 5:** Correlation of the variates included in the survey with changes in sleeping behavior based on multi-factor logit regression when corrected for gender, age, race, and marital status.

Variate		Sleeping less	Sleeping more	Sleep disturbed
Lifestyle	Student employment status	*0.056*	0.189	0.142
	Class	0.230	0.138	0.157
	Discipline	*0.065*	*0.058*	*0.080*
	International student	0.744	0.422	0.209
	Diagnosed with a sleeping problem	**0.044**	0.297	**0.035**
	Hours slept per day	**0.001**	**0.001**	**0.027**
	Frequency of feeling sleepy	**0.039**	** < 0.001**	*0.062*
	Exercise status	0.447	0.958	0.667
	Exercise level	0.463	0.597	0.281
	Balanced diet	**0.014**	0.478	0.174
	Change in diet	*0.091*	**0.002**	** < 0.001**
	BMI category	*0.095*	0.683	0.415
Living arrangement	Housing type	**0.015**	0.116	**0.001**
	Number of people living together	**0.022**	0.103	0.314
	Have children or not	*0.091*	0.115	0.330
	Adults age 60 or above in household	0.471	0.182	0.258
	Annual household income	0.161	0.137	0.147
Alcohol and tobacco use	Alcohol consumption	0.340	0.596	0.418
	Alcohol consumption frequency	0.601	0.533	*0.058*
	Changes in alcohol consumption	**0.035**	0.315	0.681
	Smoking status	0.153	0.558	0.829
	Tobacco intake frequency	**0.026**	0.800	0.156
	Change in smoking behavior	*0.062*	0.140	0.346
Vaccination status	Vaccine hesitant	*0.051*	**0.038**	0.425
	Vaccination status	0.804	0.743	0.638
	Doses received	0.109	0.107	*0.064*
Family COVID status	Student tested positive for COVID	0.388	0.881	0.466
	Student quarantined due to COVID	0.291	0.271	0.352
	Parent(s) had COVID	0.456	0.556	0.493
	Sibling(s) had COVID	0.240	0.517	0.819
	Spouse had COVID	0.111	0.729	0.546
	Child(ren) had COVID	*0.059*	0.515	*0.058*
	Grandparent(s) had COVID	0.394	0.301	0.907
	Family member died of COVID	0.208	0.241	0.271
Family vaccination status	Parent(s) vaccination status	*0.081*	0.625	0.999
	Sibling(s) vaccination status	0.549	0.809	0.777
	Spouse vaccination status	0.994	0.795	0.694
	Children 5 years old or older vaccination status	0.501	0.197	0.510
	Grandparent(s) vaccination status	0.526	*0.080*	0.382

Variates that are statistically significant at α = 0.05 are bolded while the additional variates that would be considered statistically significant at α = 0.10 are in italics. Variates in the strike-through font are not included due to a lack of sufficient combinations of the variate and the base demographic variates in the survey data.

### Sleeping less

The results of the multi-factor logit regression which included the base demographic variates gender, age, race, and marital status for the students who reported sleeping less compared to students who reported no change in their sleeping behavior are given in Table 6 for the variates that showed a statistically significant effect when corrected for the base demographics. Of the 39 variates in the survey, 9 are correlated with sleeping less in the multi-factor regression. Five lifestyle variates were correlated with sleeping less. Having been diagnosed with a sleeping problem (OR = 4.959, CI = 1.291–33.760), occasionally (OR = 3.756, CI = 1.145–14.494) or very often (OR = 30.768, CI = 8.891–130.138) feeling sleepy, and a decrease in diet quality (OR = 6.103, CI = 2.687–14.403) were all correlated with an increase in sleeping less. Students reporting having a balanced diet who reported sleeping less were less likely (OR = 0.416, CI = 0.206–0.832) than students who reported not having a balanced diet to report sleeping less.

**Table 6:** Correlation of sleeping less and selected variates when corrected for gender, age, race, and marital status including the odds ratio (OR), the corresponding lower and upper confidence levels (95% CI), and the p-value from the logit regression.

Variate		Level	Logit regression results		
			OR	(95% CI)	p-value
Lifestyle	Diagnosed sleeping problem	No (reference)	–	–	–
		Yes	**4.959**	**(1.291, 33.76)**	**0.044**
	Hours slept per day	6 h or less (reference)	–	–	–
		7–8 h	**0.04**	**(0.014, 0.101)**	** < 0.001**
		9 h or more	**0.016**	**(0.001, 0.132)**	**0.001**
	Frequency of feeling sleepy	Not at all (reference)	–	–	–
		Occasionally	**3.756**	**(1.145, 14.494)**	**0.039**
		Very often	**30.768**	**(8.891, 130.138)**	** < 0.001**
	Balanced diet	No (reference)	–	–	–
		Yes	**0.416**	**(0.206, 0.832)**	**0.014**
	Change in diet	No (reference)	–	–	–
		Yes, quality has decreased	**6.103**	**(2.687, 14.403)**	** < 0.001**
		Yes, quality has increased	2.279	(0.889, 6.012)	0.091
Living arrangement	Housing type	Rent apt or house (reference)	–	–	–
		Share apt or house	0.566	(0.16, 2.031)	0.376
		Own house	**0.238**	**(0.073, 0.731)**	**0.015**
		Other	0.586	(0.213, 1.533)	0.286
	Number of people living together	1 (reference)	–	–	–
		2	**3.294**	**(1.223, 9.372)**	**0.022**
		3	2.243	(0.846, 6.215)	0.112
		4	**3.789**	**(1.090, 14.940)**	**0.045**
		More than 4	2.184	(0.722, 7.132)	0.179
		None	2.316	(0.693, 8.539)	0.186
Alcohol and tobacco use	Changes in alcohol consumption	No (reference)	–	–	–
		Yes, decreased	**0.239**	**(0.059, 0.868)**	**0.035**
		Yes, increased	1.421	(0.685, 3.016)	0.351
	Tobacco intake frequency	Not at all (reference)	–	–	–
		Yes, daily	**3.536**	**(1.227, 11.409**)	**0.026**
		Yes, less than daily	2.017	(0.853, 5.092)	0.122

*P*-values that are statistically significant at α = 0.05 are bolded.

Living arrangements were also correlated with sleeping less. Students who lived in their own homes were less likely to report sleeping less (OR = 0.238, CI = 0.073–0.731) than students who rented an apartment or house. The number of people in the household was also correlated with sleeping less; students with two additional household members (OR = 3.294, CI = 1.223–9.372) or with four additional household members (OR = 3.789, CI = 1.090–14.940) were more likely to report sleeping less. Other forms of housing arrangements, such as subletting or temporary housing, were also more likely to report sleeping less as a collective group. However, due to the small survey size, the observed increase could not be determined to be statistically significant at the α = 0.05 level. A decrease in alcohol consumption was correlated with a decrease (OR = 0.239, CI = 0.059–0.868) in reporting sleeping less while daily intake of tobacco was correlated with an increase (OR = 3.536, CI = 1.227–11.409) in sleeping less. Less than daily intake of tobacco was also correlated with an increase in sleeping less but not at a statistically significant level, again perhaps due to the survey sample size.

### Sleeping more

The results of the multi-factor logit regression which included the base demographic variates gender, age, race, and marital status for the students who reported sleeping more compared to students who reported no change in their sleeping behavior are given in Table 7 for the variates that showed a statistically significant effect when corrected for the base demographics. Of the 39 variates in the survey, only 4 are correlated with sleeping more in the multi-factor regression.

**Table 7:** Correlation of sleeping more and selected variates when corrected for gender, age, race, and marital status including the odds ratio (OR), the corresponding lower and upper confidence levels (95% CI), and the p-value from the logit regression.

Variate		Level	Logit regression results		
			OR	(95% CI)	p-value
Lifestyle	Hours slept per day	6 h or less (reference)	–	–	–
		7–8 h	1.29	(0.441, 3.877)	0.644
		9 h or more	**11.164**	**(2.923, 51.600)**	**0.001**
	Frequency of feeling sleepy	Not at all (reference)	–	–	–
		Occasionally	0.998	(0.285, 3.698)	0.997
		Very often	**11.920**	**(3.497, 47.524)**	** < 0.001**
	Change in diet	No (reference)	–	–	–
		Yes, quality has decreased	**4.520**	**(1.773, 12.158)**	**0.002**
		Yes, quality has increased	2.168	(0.732, 6.626)	0.168
Vaccination status	Vaccine hesitant	No (reference)	–	–	–
		Yes	**2.488**	**(1.083, 6.002)**	**0.038**

*P*-values that are statistically significant at α = 0.05 are bolded.

Students who reported sleeping 9 or more hours per day (OR = 11.164, CI = 2.923–51.600), very often feeling sleepy (OR = 11.920, CI = 3.497–47.524), their diet changing and the quality decreasing (OR = 4.520, CI = 1.773–12.158), or who were hesitant to be vaccinated (OR = 2.488, CI = 1.083–6.002) were all more likely to report sleeping more than students who reported no change in their sleeping behavior.

### Disturbed sleep

The results of the multi-factor logit regression which included the base demographic variates gender, age, race, and marital status for the students who reported disturbed sleep compared to students who reported no change in their sleeping behavior are given in Table 8 for the variates that showed a statistically significant effect when corrected for the base demographics. Of the 39 variates in the survey, only five are correlated with disturbed sleep in the multi-factor regression.

**Table 8:** Correlation of disturbed sleep and selected variates, when corrected for gender, age, race, and marital status including the odds ratio (OR), the corresponding lower and upper confidence levels (95% CI),), and the p-value from the logit regression.

Variate		Level	Logit regression results		
			OR	(95% CI)	p-value
Lifestyle	Diagnosed with a sleeping problem	No (reference)	–	–	–
		Yes	**5.590**	**(1.419, 41.125)**	**0.035**
	Hours slept per day	6 h or less (reference)	–	–	–
		7–8 h	**0.444**	**(0.214, 0.899)**	**0.027**
		9 h or more	0.42	(0.114, 1.667)	0.197
	Frequency of feeling sleepy	Not at all (reference)	–	–	–
		Occasionally	*2.573*	(0.984, 7.184)	*0.062*
		Very often	**13.303**	**(4.863, 39.735)**	** < 0.001**
	Change in diet	No (reference)	–	–	–
		Yes, quality has decreased	**4.037**	**(1.922, 8.738)**	** < 0.001**
		Yes, quality has increased	1.721	(0.737, 4.134)	0.216
Living arrangement	Housing type	Rent apt or house (reference)	–	–	–
		Share apt or house	0.541	(0.17, 1.751)	0.298
		Own house	**0.149**	**(0.045, 0.45)**	**0.001**
		Other	0.455	(0.169, 1.164)	0.109

*P*-values that are statistically significant at α = 0.05 are bolded.

Students reporting a previously diagnosed sleeping problem (OR = 5.590, CI = 1.419–41.125), who very often felt sleepy (OR = 13.303, CI = 4.863–39.735), or had a change in their diet that resulted in a decrease in the quality of their diet (OR = 4.037, CI = 1.922–8.738) were all more likely to report an increase in disturbed sleep. On the other hand, students who reported sleeping 7–8 h per night were less likely (OR = 0.444, CI = 0.214–0.899) to report disturbed sleep. Students who lived in their own houses were also less likely (OR = 0.149, CI = 0.045–0.450) to report having disturbed sleep.

## Discussion

Studies have shown that infectious diseases, such as SARS, have been linked to depression, anxiety, stress, and post-traumatic stress disorder^14–17^. Traumatic events, including those caused by COVID-19, can lead to psychological distress and anxiety symptoms that can have negative impacts on sleep quality^18^. This study specifically analyzed how COVID-19 affected sleep among the NMSU students in New Mexico, USA. Among university students and administration staff in Milan, Italy, bedtime hours, wake-up time, and sleep latency all increased from before to during COVID-19. Particularly in students, the delay in bedtime and wake-up was more pronounced. A worsening of sleep quality and insomnia symptoms were also found. During COVID-19 bedtime was delayed 39 min for students and wake-up was delayed 64 min. A significant increase in sleep problems was found between before and during COVID-19 as well as clinical insomnia. For maintenance of insomnia and early morning awakening, 20% showed problems before COVID-19 and 30% after COVID-19^4^. Furthermore, in a study of 195 university students in Texas, USA, 86% reported disruptions to their sleep patterns caused by the COVID-19 pandemic, with 38% of them reporting the disruption as severe. 50% who reported a disruption tended to stay up later or wake up later than they did before COVID-19. Also noted were irregular sleep patterns, including inconsistent times going to bed and waking up from day to day. Nearly, 7% students reported an increase in sleep hours due to less outdoor activities, while 6% had poor sleep quality^9^. In this study, being diagnosed with a sleeping problem was correlated with increased odds of disturbed sleep and sleeping less. Among hours slept, sleeping 7–8 h per day was protective against disturbed sleep.

Further studies analyzed additional aspects of COVID’s effect on sleep in populations beyond university students. Among 207 nursing students assessed before and during the pandemic, the Pittsburg Sleep Quality Index (PSQI) score was on average 0.91 points worse during the lockdown (95% CI =  − 0.51, − 1.31). Other significant changes included lower sleep quality, higher sleep latency, decreased sleep duration, increased sleep disturbance, and later bedtime and later wake-up times. When stratified by demographic characteristics, PSQI scores were significantly lower during the lockdown in women, normal weight, first-year students, second-year students, alcohol drinkers, and living with parents. In addition, smokers experienced a reduction in sleep quality of 2.29 points compared to non-smokers, and students with anxiety/depression had a reduction in sleep quality of 1.74 points^19^. This study found similar trends in the impact of sleep patterns. Decreased alcohol consumption during the pandemic was correlated with a protective factor for sleeping less, and alcohol use 2–4 times per month was correlated with an increased risk of disturbed sleeping. This finding could be due to restricted or limited access to purchase alcohol during the lockdown during COVID-19, which is correlated with individuals leading healthier lifestyles and improved sleeping quality. Individuals who consumed alcohol 1–4 times per month who showed an increased risk for disturbed sleeping could be due to shifting from regular alcohol consumption that may affect normal sleeping hours. Daily tobacco intake had increased odds for sleeping less. This finding could be due to the limited or restricted purchase of tobacco and its effect on sleep patterns due to stress correlated with tobacco consumption. These findings required further research.

Among demographics, our study found several significant correlation between age and sleep. Marelli et al.^4^ found that older subjects had lower total sleep time, bedtime, wake-up, and insomnia severity index (ISI) scores. Older participants also had significantly lower scores in somatic-affective Beck Depression Inventory (BDI), cognitive BDI, and total BDI, although the age range was not defined. Among the general population, Tasnim et al.^20^ found that young people (ages 18–34) reported sleep problems more frequently than elderly people, 79% vs. 72% in ages ≥ 35 years. In this analysis, ages 30–39 years old were correlated with a protective factor for sleeping less.

Although this study analyzed a population of college students, there is limited literature analyzing induvial covariates and their correlation with sleep during COVID-19. Through a review of 78 studies, Tselebis et al.^21^ identified several covariates that were correlated with higher risk of insomnia including female gender, level of education, preexisting disease, reduced physical activity, and higher income^21^. Furthermore, women had worse total scores than men^4^. In our study, males were correlated with a significant protective factor in both sleeping less and sleeping more. Family support has been evaluated using the Family Support Scale (FSS), which determined the sense of support from members of a participant’s family and friends using 13 likert questions. FSS was negatively correlated the Athen’s Insomnia Scale (AIS) in nursing staff during COVID-19^22^. This study did not directly have a questionnaire on family support but included another type of housing arrangement, which included family support to the student during COVID-19. In this study, we evaluated family support through several covariates including marital status and number of children. A status of married was correlated with a protective factor for sleeping less, sleeping more, and disturbed sleeping. Owning a house was protective for sleeping less and disturbed sleep due to uncertainty with financial assistance students received as research or teaching assistant during the COVID-19 era from their affiliated schools. Among number of people living together, two and four were correlated with an increased odds of sleeping less; however, having three children was correlated with a protective factor for sleeping less. Overall, most factors providing support led to protective Correlation with sleeping changes. Chen et al.^23^ also identified that among the number of people living together, caring for patients with COVID-19 and having a sleep disorder were correlated with increased odds of insomnia. In comments of their questionnaire given, poor sleep was mapped to categories of children and family, work demands, personal health, and pandemic-related sleep disturbances^23^. Our study found that additional factors, including vaccine hesitancy, was correlated with increased odds of sleeping less. Other findings in this study included correlation between diet and changes in sleep. Those who ate a balanced diet had significant protective association with sleeping less and disturbed sleeping. Among changes in diet, decreased quality was correlated with increased odds of sleeping less, sleeping more, and disturbed sleeping. Increased quality of diet was correlated with increased odds of sleeping less, and a borderline association of sleeping more. Additionally, this study reported teaching assistants are less likely to report sleeping less than other students who are employed. One possible explanation for why teaching assistants are less likely to report sleeping less than other student employees is because their job comes with more structured schedules and regular work hours compared to other employed students. Other confounding variables not included in the analysis are workload, commute time, job flexibility, and personal responsibilities.

The population of NMSU includes a large proportion of Hispanic and minority students. Studies have found that racial/ethnic minorities and other disadvantaged populations are more vulnerable to COVID-19 due to the disproportionate burden of immune-compromising chronic conditions^24,25^. Racial and ethnic minorities are more likely to have low-wage jobs without worker protections, use public transportation, and live in substandard multifamily units^25–28^. Furthermore, this group is less likely to get physiological and social needs met such as getting adequate sleep and livable wages in addition to stressors that likely heightened due to COVID-19 including medical mistrust and less access to COVID-19 testing^25,29^. These stressors can induce fear and anxiety, which can also contribute to worsening sleep habits^25,30–32^. In this study, race and ethnicity were not directly correlated with sleep disorder but it is an underlying aspect of the population when considering equity in COVID-19’s effect on sleep.

### Limitations

This study is a cross-sectional study, which means that causation cannot be determined. The data for this study was collected through a questionnaire, relying on participants' self-reported responses rather than formal clinical diagnoses or the use of sleep recording technologies. It is important to note that due to the nature of the pandemic, there is no available control data from before the outbreak. Consequently, a direct comparison or contrast of the data collected during the pandemic with pre-pandemic data to establish differences is not possible.

Due to the study design of collecting the data at one point in time, specifically during the end of the pandemic, we were unable to make comparisons with data prior to COVID-19. Therefore, the data analysis includes several covariates, all collected during the end of the pandemic. Additionally, all data was collected through questionnaires, which limited our ability to determine aspects such as COVID-19 status or vaccination status, except through the recorded answers.

Furthermore, all data, including the number of hours slept, prior sleep disorders, and changes in sleep, was determined by participants and not through further clinical testing or the use of recording devices. Also, more detailed questions on some variables, such as a balanced diet were not included in the questionnaire due to the lengthy questionnaire on many variables.

## Conclusion

The COVID-19 pandemic led to numerous changes in university students at NMSU with respect to sleep patterns. Covariates regarding base demographics, lifestyle, living arrangement, family vaccination status, and alcohol and tobacco use were all correlated with changes in sleep habits including sleeping less, sleeping more, and disturbed sleep. These findings demonstrate the numerous mental health effects of COVID-19 on students. Further research is needed to understand the mechanisms behind specific changes in university settings. This can be achieved by investigating the impact of factors such as online classes or social isolation on sleep patterns, which may lead to changes in sleep.

## Data Availability

The data generated or analyzed during the study are available upon reasonable requested by the corresponding author (email: hrahman@nmsu.edu) and Dr. Stuart H Munson-McGee (email: sh.munsonmcgee@gmail.com).
